# Massachusetts Veterinary Association

**Published:** 1892-06

**Authors:** Austin Peters

**Affiliations:** Secretary


AUSTIN PETERS, *Secretary.*

---

*Massachusetts Veterinary Association.*—The regular meeting of the Massachusetts Veterinary Association was held at 19 Boylston Place, Boston, Wednesday evening, May 25, at 7:30 o'clock. President L. H. Howard in the chair. Members present: Drs. Blackwood, Bunker, Becket, Burr, Hadcock, Hitchings, Howard, Marshall, Osgood, Saunders, Winchester, Hitchcock, Parker, La Baw, and the Secretary. Honorary members: Drs. Stickney and A. Liautard, Dean of the Faculty of the American Veterinary College. Guests: Drs. H. D. Atwood and W. H. Brownell, both of Brockton, and Messrs. F. H. Maloney, G. F. Quinlan, A. L. Murch, James Marshall and S. O. Fowle, senior students at the Harvard Veterinary School.

Records of the last meeting read and accepted. Committee Reports: Dr. Winchester reports for the Committee of the Association to arrange for the entertaining of the United States Veterinary Medical Association next September, that the best way of doing so, perhaps, would be to arrange for a sail down the harbor, with a collation on board, the expense to be met by voluntary subscription on the part of the members. He had therefore prepared a subscription paper for those who desired to do so to sign. Dr Bunker, as a member of the Committee to arrange for invited guests, said he had not come prepared to make a final report, but thought that the chairmen of some of the Boards of Health in our larger cities should be invited.

Dr. Saunders, as the member of the Committee to inquire into the expense of chartering a boat for the day, reported upon what he had done. A general discussion by various members upon arrangements for the occasion followed.

Admission of members: The name of F. B. Carlton, M. D. V., of Salem, was balloted upon. Fifteen votes were cast, all in the affirmative, and Dr. Carlton was therefore declared elected Application for membership: Wm. Stinson, D. V. S., of Chelsea, filed his application with the Secretary, Dr. W. A. Hitchcock presenting his credentials. Dr. Osgood, as Chairman of the Executive Committee, reported that the credentials were all right, and Dr. Stinson's name was laid upon the table, under the rules, to be balloted upon at the June meeting.

New business: Dr. Osgood moves that an auxiliary Reception Committee of ten be appointed by the chair to assist in taking care of our guests next September. Seconded by Dr. Bunker. Carried. Later in the evening the President announced the following members of the Association to serve upon this committee: Drs. F. H. Osgood, Chairman; Thomas Blackwood, W. Bryden, E. C. Becket, W. H. Hitchings, Alex. Burr, Chas. E. Hadcock, Daniel Emerson, W. A. Hitchcock and John M. Parker.

In reply to a question by Dr. Bunker, Dr. Winchester said that Dr. Howard and himself. together with Dr. Hoskins, of Philadelphia, were a Committee from the U. S. V. M. A. to arrange for the meeting. and that the work of procuring a hall devolved upon them, and not upon any Committee of the Massachusetts Veterinary Association.

Dr. Liautard, the honored guest of the evening, then read a very interesting paper upon "Professional Ethics." Dr. Marshall's views agreed with Dr. Liautard's.

Dr. Osgood endorsed the paper, thinks we ought to have a three-years' graded course, and a standard of veterinary education, recognized everywhere. He did not think that we could get a uniform standard in all the schools, but thinks that certain schools, recognized as *par excellence*, might create a standard.

Dr. Parker thinks that we should do all in our power to elevate the profession, and agrees with Dr. Osgood in his views.

Dr. Winchester thinks that in the Massachusetts Veterinary Association the members have, in most instances, a proper idea of what professional ethics ought to be. That is, in most respects, our members stand well in point of ethical behavior. He also thinks that the elevation of the profession depends largely upon the members as individuals.

Dr. Liautard said that in looking over the Union, he thinks the students are not so much to blame for the condition of things as are some of the older men, in the way they behave, and the example they set. If we set about securing a National Board of Examiners, he thinks the interstate law may be the hardest thing to overcome, but thinks it best to try and do something while the profession is young and its members are few. It may take years to bring about the change, We can do more while we have only ten schools, than by waiting until we have twenty, or perhaps two in every State.

Dr. Osgood is afraid we cannot get the same protection granted by every State to members of the profession; that is why he thinks graduates of different schools will carry different weights.

Dr. Winchester thinks that everything must have a beginning, and that any advance is generally brought about by a few men. He also believes that some schools, by means of many teachers, can teach more in two years than others with a few instructors can teach in three or four years. Dr. Osgood asks if more time is not desirable. Dr. Liautard thinks more time is desirable, if possible. Dr. Saunders compares students' heads upon entering college with empty spaces, and the instruction they receive with material to fill these spaces, and is of the opinion that "if you put out the stuff you will fill up the room."

Dr. Bunker likes Dr. Liautard's paper, as it has taken us out of our usual rut. He is proud to be a graduate of the American Veterinary College.

The "Frenchman" has always been square and honest and if his medicine is sometimes bitter, still it agrees with us. Dr. Bunker moves that the essayist be accorded a vote of thanks for his interesting paper. Seconded by Dr. Saunders. Carried.

Dr. Bunker then read a paper reporting several cases he had had during the winter, in his practice, as follows: Case of paralysis of the throat, in a horse; case of cancer of the liver, in a dog; case of tuberculosis of the liver, in a bull, specimen shown; case of mumified fœtus, in a cow, specimen shown.

Meeting adjourned. AUSTIN PETERS, *Secretary.*

